# Sporotrichoid cutaneous tuberculosis in the peruvian amazon: case report

**DOI:** 10.17843/rpmesp.2022.393.10919

**Published:** 2022-09-30

**Authors:** Percy Zamora-Perea, Alfrando Moreno, Eduardo Panduro-García, Marco Fabrizio Paredes-Obando, Jhosephi Jhampier Vásquez-Ascate, Freddy André Ferreyra, Edgar Antonio Ramírez-García

**Affiliations:** 1 Universidad Nacional de la Amazonía Peruana, Iquitos, Peru. Universidad Nacional de la Amazonía Peruana Universidad Nacional de la Amazonía Peruana Iquitos Peru; 2 Hospital Regional de Loreto, Iquitos, Peru. Hospital Regional de Loreto Iquitos Peru

**Keywords:** Tuberculosis, Cutaneous, *Mycobacterium tuberculosis*, Sporotrichosis, Giant Cells, Langhans

## Abstract

Cutaneous tuberculosis is a rare presentation of *Mycobacterium tuberculosis* infection. We present the case of a woman without important medical history, with a disease period of one year and a half, characterized by sporotrichoid-like lesions, with lymphocutaneous dissemination in the right upper limb, and with slowly progressive evolution. The histopathological tests revealed Langhans type giant cells and scarce necrosis. The patient received therapy with a sensitive antituberculous scheme, and evolved favorably.

## INTRODUCTION

Tuberculosis caused by *Mycobacterium tuberculosis* is the infectious disease with the highest mortality rate, after COVID-19 ^1^. Cutaneous lesions represent 1 - 1.5% of all manifestations of extrapulmonary tuberculosis, and are currently classified according to their morphological-clinical pattern, the route of acquisition and the immunological status of the host ^2^ ^,^ ^3^.

One of the main forms of infection is by “primary tuberculosis inoculation,” which occurs by exogenous inoculation in a population not previously sensitized to *M. tuberculosis* or in children not immunized against bacillus Calmette-Guérin (BCG). It starts with a nodular lesion on the face or upper or lower extremities, which eventually ulcerates after 2 or 3 weeks, and there is also non-painful lymphadenopathy associated with lymphocutaneous complex analogous to Ghon’s complex in pulmonary infection ^4^. Diagnosis is done by early biopsy, showing a neutrophilic infiltrate that evolves to produce a necrotizing tuberculous granuloma with the presence of multiple acid-fast bacilli ^5^ ^,^ ^6^.

There are few reports of cases of tuberculosis due to primary inoculation with a sporotrichoid pattern and lymphocutaneous spread, which complicates its diagnosis. We report a case of chronic skin lesions with painless shallow ulcers with granulomatous base and lymphocutaneous spread. The anatomopathological study described cutaneous tuberculosis; and therefore, antituberculosis therapy was initiated with good response.

## CASE REPORT

### Patient information

We present the case of a 14-year-old female patient from the city of Iquitos, in the Peruvian Amazon, with no medical history, no loss of appetite, and no weight loss or nocturnal temperature increase. She had 18 months of illness before hospital admission. The main symptom was an increase in volume on the dorsum of the right hand, initially painless, which progressed from a nodular lesion to an ulcerated lesion, to later develop an apparently lymphatic dissemination throughout the right upper extremity, without other accompanying symptoms. She attended the outpatient service of the dermatology department of the Regional Hospital of Loreto. She did not report contact or recent travel, neither any injury risk factors associated with the onset of signs and symptoms. She had no previous history of tuberculosis or use of immunosuppressant drugs, nor did she report any close or family contact with patients infected with tuberculosis. The patient mentioned that she went to a private clinic without a successful outcome, she did not disclose the treatment received. She did not undergo immunological tests for tuberculosis such as Mantoux test or QuantiFERON Gold test.

### Clinical findings

**Figure 1:**
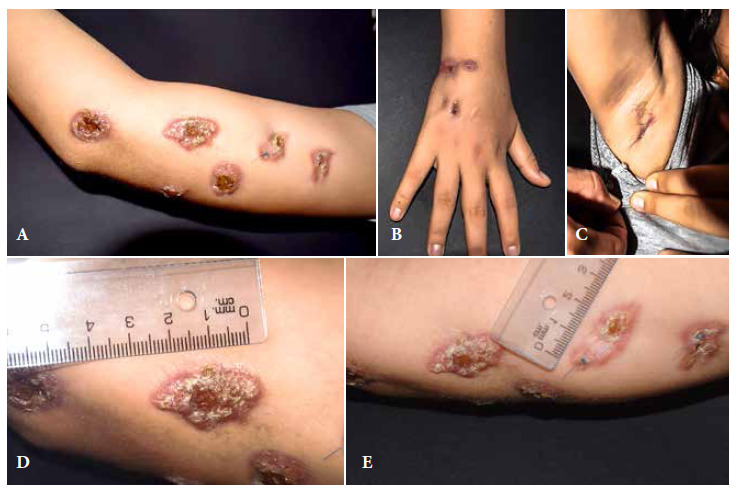
Cutaneous lesions on the right upper limb. A. Six lesions on the inner aspect of the right arm. B. Two sporotrichoid lesions on the dorsal region of the right hand. C. Scrofuloderma on the inner region of the right armpit. D. Larger lesion on the inner aspect of the right arm, 3 cm long, sporotrichoid-like with erythematous borders, presence of clear scabs with ulcerative center. E. Stitched lesion on the inner aspect of the right arm where the biopsy was taken.

During physical examination, a lesion with erythematous, soft bottom extending over the dorsum of the hand was found on the inner side of the right arm. Serosanguinous secretion was observed after incision. In addition, there was evidence of ulcers with raised, crusty and everted edges with presence of granulation tissue at the base (Figure 1). No regional lymphadenopathy was found by palpation, only a scrofuloderma lesion was evidenced in the right armpit.

### Timeline

Three weeks after the intervention, a violaceous nodular lesion of 22 x 22 mm in diameter was observed on the inner side of the arm of the affected limb; after another three weeks, the primary nodule formed an ulcer with seropurulent secretion. Subsequently, new lesions of similar characteristics developed in the same area of the arm, with linear course throughout the limb, with periods of 2 to 3 weeks between them; one of them appeared on the wrist and others laterally. After one year, another one appeared on the inner elbow. Overall, there were ten cutaneous lesions with lymphocutaneous spread over the entire right upper extremity.

### Diagnostic evaluation

**Figure 2:**
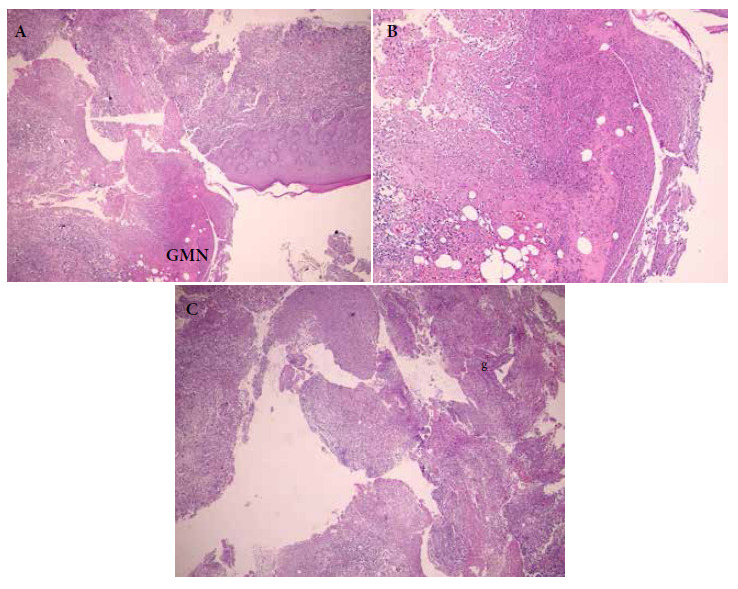
Skin biopsy of the lesion in the anteromedial region of the right arm. A. Ulcerous bed, and area of reactive epidermis. B. Ulcerous bed with extensive necrosis, extension to deep dermis. C. Ulcerous bed, presence of granulomas (g), giant multinucleated cell (GMN).

The patient received antibiotic and antifungal treatment without any improvement. Routine hemogram and biochemical tests were normal. Indirect immunofluorescence test for leishmaniasis was performed, with negative results. In addition, the chest X-ray did not show any alteration. Potassium hydroxide examination of the skin lesions and cultures for bacterial and fungal microorganisms were negative. The biopsy report (Figure 2) revealed a sample of epidermis with irregular and papillomatous acanthosis. The report showed superficial and deep dermis with abundant granulomatous inflammatory infiltrate with presence of macrophages, lymphocytes, and plasma cells. Langhans type giant cells and scarce necrosis were present. Ziehl-Neelsen staining was negative, Giemsa staining was not contributory. Histopathological findings were consistent with cutaneous tuberculosis.

### Therapeutic intervention

Treatment was initiated with a first-line drug-sensitive antituberculosis scheme: pyrazinamide 900 mg; ethambutol 700 mg; rifampicin 350 mg, and isoniazid 175 mg/day. 

### Follow-up and results

Currently the patient continues to receive antituberculosis therapy and with apparent resolution of the lesions.

## DISCUSSION

Cutaneous tuberculosis simulating sporotrichosis differs from other types of subcutaneous tuberculosis, such as scrofuloderma, because of an apparent clinically underlying tuberculous site. This site was not found in the case we are presenting. Besides, sporotrichosis-type cutaneous tuberculosis can be observed in children and young people ^8^ ^,^ ^9^. The initial diagnosis of this condition, made by biopsy, shows the presence of high loads of acid-alcohol-fast-bacilli (AAFB), however, in long cases, these bacilli decrease, with presence of epithelioid granuloma and Langhans type giant cells ^5^ ^,^ ^10^ ^-^ ^12^.

Sometimes the special Ziehl-Neelsen staining of sputum samples does not reveal the presence of the microorganism in the sample ^8^ ^-^ ^9^ ^,^ ^13^ ^-^ ^15^, as in this case. Lesions can evolve for a long time and will continue to appear if treatment is not initiated. The cutaneous lesions diminish in response to specific treatment for tuberculosis, with complete resolution after five months of anti-tuberculosis therapy; however, there may be a rapid evolution after two months of starting this therapy ^9^ ^,^ ^14^ ^,^ ^16^. In our case, the patient continues in the second phase of antituberculosis therapy with a sensitive scheme, with resolution of the primary site and without new lesions.

The mechanism of infection in this case is unknown and primary inoculation of tuberculosis is suspected, because of the localization in upper and lower limbs. This unusual presentation is accompanied by lymphatic spread, with exogenous inoculation ^7^ ^,^ ^9^.

One of the limitations of this report is that molecular tests for the identification of *Mycobacterium tuberculosis* were not performed, nor immunological tests such as the Mantoux test or the QuantiFERON Gold test. These tests were not available at the Regional Hospital of Loreto. In addition, no cultures were performed by the tuberculosis program of the hospital.

In conclusion, the clinical presentation of cutaneous tuberculosis should be considered during the differential diagnosis of sporotrichosis-like skin lesions, especially in patients with no antibiotic or antifungal response, and in areas with a high incidence of tuberculosis.

## References

[1] Organización Mundial de la Salud (2021). Tuberculosis.

[2] Franco-Paredes C, Marcos LA, Henao-Martínez AF, Rodríguez-Morales AJ, Villamil-Gómez WE, Gotuzzo E (2018). Cutaneous Mycobacterial Infections. Clin Microbiol Rev.

[3] van Zyl L, du Plessis J, Viljoen J (2015). Cutaneous tuberculosis overview and current treatment regimens. Tuberc Edinb Scotl.

[4] Bravo FG, Gotuzzo E (2007). Cutaneous tuberculosis. Clin Dermatol.

[5] Afsar FS, Ozcelik S, Uysal SS, Ermete M, Afsar I (2015). Primary inoculation tuberculosis a report of a rare entity. Rev Soc Bras Med Trop.

[6] Cardona-Hernández MÁ, González-González M, Cruz FJ-S, Romero-Guzmán AK (2021). Tuberculosis cutánea recurrente, variedad nodular profunda Comunicación de un caso y revisión de la literatura. Rev Cent Dermatológico Pascua.

[7] Belchior I, Seabra B, Duarte R (2011). Primary inoculation skin tuberculosis by accidental needle stick. BMJ Case Rep.

[8] Remenyik E, Nagy B, Kiss M, Veres I, Sápy M, Horkay I (2005). Sporotrichoid cutaneous Mycobacterium tuberculosis infection in a child. Acta Derm Venereol.

[9] Premalatha S, Rao NR, Somasundaram V, Razack EMA, Muthuswami TC (1987). Goma tuberculosa en patrón esporotricoides. Int J Dermatol.

[10] Nagesh TS, Akhilesh A (2014). Sporotrichoid cutaneous tuberculosis. Indian J Dermatol Venereol Leprol. junio de.

[11] Ramesh V (2007). Sporotrichoid cutaneous tuberculosis. Clin Exp Dermatol. noviembre de.

[12] Pau WSC, AlSaffar H, Weinstein M, Kitai I (2009). Sporotrichoid-like tuberculosis. Pediatr Infect Dis J.

[13] Downey C, Navajas L, Andino R, Vera-Kellet C, Manríquez JJ (2015). Sporotrichoid-like tuberculosis: an unusual presentation of cutaneous tuberculosis in an immunocompetent patient. Rev Chil Infectologia Orga.

[14] Nakamura S, Hashimoto Y, Nishi K, Takahashi H, Takeda K, Mizumoto T (2012). Cutaneous tuberculosis simulating lymphocutaneous sporotrichosis. Australas J Dermatol.

[15] Göktay F, Aydingöz IE, Mansur AT, Cobanoglu MF, Cavusoglu C (2007). Detección del complejo Mycobacterium tuberculosis mediante ensayo de sonda lineal en un caso con lesiones cutáneas esporotricoides. J Eur Acad Dermatol Venereol.

[16] Hadj I, Meziane M, Mikou O, Inani K, Harmouch T, Mernissi FZ (2014). Tuberculous gummas with sporotrichoid pattern in a 57-year-old female A case report and review of the literature. Int J Mycobacteriology.

