# Supplementary material for: Sporotrichoid cutaneous tuberculosis in the peruvian amazon: case report
Source: Rev Peru Med Exp Salud Publica. 2022 Sep 30;39(3):368–71. doi: 10.17843/rpmesp.2022.393.10919 (PMC11397584; doi:10.17843/rpmesp.2022.393.10919)
Supplement: Translation

# Translation: Tuberculosis cutánea esporotricoide en la Amazonía peruana: reporte de caso

**Authors:** Percy Zamora-Perea, Alfrando Moreno, Eduardo Panduro-García, Marco Fabrizio Paredes-Obando, Jhosephi Jhampier Vásquez-Ascate, Freddy André Ferreyra, Edgar Antonio Ramírez-García

**Affiliations:** 1 Universidad Nacional de la Amazonía Peruana, Iquitos, Perú.; 2 Hospital Regional de Loreto, Iquitos, Perú.

**Keywords:** Tuberculosis Cutánea, *Mycobacterium tuberculosis*, Esporotricosis, Células Gigantes de Langhans

## Abstract

La tuberculosis cutánea es una presentación rara de la infección por *Mycobacterium tuberculosis*. Se presenta el caso de una mujer sin antecedentes médicos de importancia, con un tiempo de enfermedad de año y medio, caracterizado por lesiones tipo esporotricoide, con diseminación linfocutánea en miembro superior derecho, de evolución lentamente progresiva. Se realizó un estudio histopatológico encontrándose células gigantes tipo Langhans y escasa necrosis. El paciente recibió terapia de esquema sensible antituberculoso, con evolución favorable.

## INTRODUCCIÓN

La tuberculosis causada por *Mycobacterium tuberculosis* es la enfermedad infecciosa de mayor mortalidad, luego de la COVID-19 ^1^. Las lesiones cutáneas representan el 1 - 1,5% de todas las manifestaciones de tuberculosis extrapulmonar, actualmente se clasifican por su patrón morfológico-clínico, la vía de adquisición y el estado inmunológico del hospedador ^2^ ^,^ ^3^.

Una de las principales formas de infección es por «inoculación primaria de tuberculosis», que sucede por inoculación exógena en población no sensibilizada previamente a *M. tuberculosis* o en niños no inmunizados contra el bacilo de Calmette-Guérin (BCG). Se presenta con lesión nodular en la cara o en las extremidades superiores o inferiores, que eventualmente se ulcera luego de 2 o 3 semanas, además se presenta linfadenopatía no dolorosa asociada a complejo linfocutáneo análogo al complejo de Ghon en infección pulmonar ^4^. El diagnóstico es por biopsia temprana, donde se muestra un infiltrado neutrofílico que evoluciona para producir un granuloma tuberculoso necrosante con presencia de múltiples bacilos ácido alcohol resistentes (BAAR) ^5^ ^,^ ^6^ ^).^.

Existen pocos informes de casos de tuberculosis por inoculación primaria, que tengan un patrón esporotricoide con recorrido linfocutáneo, por lo que su diagnóstico es complicado. Se reporta un caso de lesiones cutáneas crónicas de tipo úlceras indoloras poco profundas con base granulomatosa con recorrido linfocutáneo, cuyo estudio anatomopatológico describió una tuberculosis cutánea, por lo que se le inició terapia antituberculosa con buena respuesta.

## REPORTE DE CASO

### Información del paciente

Paciente escolar femenina de 14 años, procedente de la ciudad de Iquitos, en la Amazonía peruana. Sin antecedentes médicos, ni pérdida de apetito, sin pérdida de peso o aumento nocturno de la temperatura. Con tiempo de enfermedad de 18 meses antes del ingreso hospitalario, refiere como principal síntoma el aumento de volumen en el dorso de la mano derecha, indolora inicialmente, que progresa de lesión nodular a lesión ulcerada, para posteriormente desarrollar una diseminación aparentemente linfática en toda la extremidad superior derecha, sin otros síntomas acompañantes. Acude a consulta de atención externa en el servicio de dermatología del Hospital Regional de Loreto. No refiere ningún factor de riesgo traumático, de contacto o viajes recientes, asociados con la aparición de signos y la sintomatología. No presenta historia previa de tuberculosis o uso de fármacos inmunosupresores, tampoco refiere algún contacto cercano o familiar con pacientes infectados con tuberculosis. La paciente menciona que acudió a un consultorio privado sin resultado exitoso, no dio a conocer el tratamiento recibido. No se realizó exámenes inmunológicos para tuberculosis como la prueba de Mantoux o la prueba de QuantiFERON Gold.

### Hallazgos clínicos

Al examen físico, en la cara interna de brazo derecho presentó lesión con fondo eritematoso, blando, que se extiende sobre el dorso de la mano, que tras la incisión se obtiene secreción serosanguinolenta. Además, se evidencia úlceras con bordes elevados costrosos y evertidos con presencia de tejido de granulación en las bases (Figura 1). No se observó adenopatías regionales a la palpación, solo se evidenció una lesión escrofuloderma en axila derecha.

**Figura 1:** Lesiones cutáneas en miembro superior derecho. **A. **Seis lesiones en cara interna del brazo derecho. **B. **Dos lesiones de aspecto esporotricoide en región dorsal de mano derecha. **C. **Escrofuloderma en región interna de axila derecha. **D. **Lesión de mayor tamaño en cara interna de brazo derecho, 3 cm de longitud, aspecto esporotricoide con bordes eritematosos, presencia de escaras claras con centro ulcerativo. **E. **Lesión con suturas en cara interna de brazo derecho de donde se obtuvo la biopsia.

### Cronología

A las tres semanas luego de la intervención, se apreció una lesión nodular violácea de 22 x 22 mm de diámetro en cara interna del brazo del miembro afectado; tras otras tres semanas el nódulo primario formó una úlcera con secreción seropurulenta. Consiguientemente, se desarrollaron nuevas lesiones de características similares en misma zona del brazo, con recorrido lineal en toda la extremidad, en períodos de 2 a 3 semanas entre ellas; una de ellas apareció en la muñeca y otras más lateralmente. Al cabo de un año, apareció otra en la flexura del codo. En general, son diez lesiones cutáneas con un recorrido linfocutáneo en toda la extremidad superior derecha.

### Evaluación diagnóstica

Recibió tratamiento antibiótico y antifúngico sin mejoría alguna de las lesiones. Los exámenes de hemograma y bioquímico de rutina fueron normales. Se realizó la prueba de inmunofluorescencia indirecta para leishmaniasis, con resultados negativos. Además, la radiografía de tórax no evidenció ninguna alteración. El examen de hidróxido de potasio en lesión de piel y cultivos para microorganismos bacterianos y fúngicos fue negativo. El informe de biopsia (Figura 2) revela muestra de epidermis con acantosis irregular y papilomatoso. Dermis superficial y profunda con abundante infiltrado inflamatorio granulomatoso con presencia de macrófagos, linfocitos y células plasmáticas. Presencia de células gigantes tipo Langhans y escasa necrosis. A la tinción con Ziehl-Neelsen se obtuvo resultado negativo, tinción Giemsa no contributario. Los hallazgos histopatológicos son consistentes con tuberculosis cutánea.

**Figura 2:** Biopsia de piel de la lesión de región anteromedial del brazo derecho. **A**. Lecho ulceroso, y zona de epidermis reactiva. **B. **Lecho ulceroso con extensa necrosis, extensión a dermis profunda. **C. **Lecho ulceroso. g: presencia de granulomas, GMN: célula gigante multinucleada.

### Intervención terapéutica

Se inició tratamiento con abordaje de esquema antituberculoso sensible a drogas de primera línea: pirazinamida 900 mg; etambutol 700 mg; rifampicina 350 mg, e isoniazida 175 mg/día. 

### Seguimiento y resultados

En la actualidad la paciente continúa recibiendo terapia antituberculosa y se puede observar resolución aparente de las lesiones.

## DISCUSIÓN

La tuberculosis cutánea que simula la esporotricosis se diferencia de otras tuberculosis subcutáneas, como la escrofuloderma, por un aparente foco tuberculoso subyacente clínicamente. En el presente caso no se evidenció dicho foco, además que la tuberculosis cutánea de tipo esporotricoide se puede observar en población infantil y en jóvenes ^8^ ^,^ ^9^ cuyo diagnóstico inicial con biopsia es la presencia de alta carga de bacilos ácidos alcohol resistente (BAAR), pero en casos de larga evolución estos bacilos disminuyen, con observación de granuloma epitelioide con células gigantes tipo Langhans ^5^ ^,^ ^10^ ^-^ ^12^. 

Algunas veces la coloración especial de Ziehl-Neelsen en muestras de esputo no revela la presencia del microorganismo en la muestra ^8^ ^-^ ^9^ ^,^ ^13^ ^-^ ^15^, como se dio en este caso. Las lesiones pueden evolucionar durante mucho tiempo y continuarán apareciendo mientras no se inicie tratamiento. Las lesiones cutáneas disminuyen en respuesta al tratamiento específico para tuberculosis, teniendo resolución completa al cabo de cinco meses de terapia antituberculosa; sin embargo, puede darse una rápida evolución a los dos meses de esta terapia ^9^ ^,^ ^14^ ^,^ ^16^. En nuestro caso la paciente continua en segunda fase de terapia antituberculosa de esquema sensible, con resolución de foco primario y sin evidenciarse nuevas lesiones.

Se desconoce el mecanismo de infección del caso descrito y se sospecha de la inoculación primaria de tuberculosis, cuya característica se da por localización en extremidades superiores e inferiores. Esta presentación inusual se acompaña del recorrido linfático, con inoculación exógena ^7^ ^,^ ^9^.

Entre las limitantes del caso, se debe mencionar que no se realizó pruebas moleculares para identificación de *Mycobacterium tuberculosis*, ni pruebas inmunológicas como la prueba de Mantoux o la Prueba de QuantiFERON Gold; dichas pruebas no se encuentran a disposición del establecimiento de salud mencionado. Además, no se realizaron cultivos por parte del programa de tuberculosis de dicho establecimiento. 

En conclusión, la presentación clínica de la tuberculosis cutánea debe tenerse en cuenta en el diagnóstico diferencial de las lesiones cutáneas similares a la esporotricosis, especialmente en pacientes sin respuesta antibiótica o antifúngica, y en zonas de alta incidencia de tuberculosis.
